# Association between pelvic floor muscle strength and sexual function based on PISQ-12—an analysis of data from a multicenter cross-sectional study on 735 nulliparae during pregnancy

**DOI:** 10.3389/fmed.2023.1093830

**Published:** 2023-04-25

**Authors:** Lei Gao, Bing Xie, Hongmei Zhu, Di Zhang, Xiuhong Fu, Hongjuan Li, Min Zhen, Baoling Qin, Weipeng Chen, Xuying Mao, Lingrui Kong, Jianliu Wang, Guizhu Wu, Xiuli Sun

**Affiliations:** ^1^Department of Obstetrics and Gynecology, Peking University People's Hospital, Beijing, China; ^2^The Key Laboratory of Female Pelvic Floor Disorders, Peking University People's Hospital, Beijing, China; ^3^Department of Sports Medicine and Rehabilitation, Beijing Sports University, Beijing, China; ^4^Department of Obstetrics and Gynecology, Henan Key Laboratory of Fertility Protection and Aristogenesis, Luohe, China; ^5^Department of Obstetrics and Gynecology, Luohe Central Hospital of Henan Province, Luohe, China; ^6^Department of Obstetrics and Gynecology, Zhengzhou Central Hospital Affiliated to Zhengzhou University, Zhengzhou, China; ^7^Department of Obstetrics and Gynecology, Fangshan District Beijing Maternal and Child Health Hospital, Beijing, China; ^8^Department of Obstetrics and Gynecology, Beijing Fengtai District Maternal and Child Health Hospital, Beijing, China; ^9^Department of Obstetrics and Gynecology, Peking University International Hospital, Beijing, China; ^10^Department of Obstetrics and Gynecology, Peking University Shenzhen Hospital, Shenzhen, China; ^11^Department of Obstetrics and Gynecology, Capital Medical University Mentougou Teaching Hospital, Beijing, China; ^12^Department of Obstetrics and Gynecology, Shanghai First Maternity and Infant Hospital, School of Medicine, Tongji University, Shanghai, China

**Keywords:** muscle strength (MeSH), pelvic floor (MESH unique ID = D017773), pregnant women, sexual dysfunction, women

## Abstract

**Background:**

Pelvic floor muscle strength is well-known to be associated with female sexual function. However, there were a few studies that reported on the relationship between pelvic floor muscle strength and female sexual function in pregnant women, and the presented results were inconsistent. Nulliparae represent a specific cohort with simplicity to exclude confounding factors that are caused by parity. The present study aimed to explore the association of pelvic floor muscle strength and sexual function based on the Pelvic Organ Prolapse/Urinary Incontinence Sexual Questionnaire (PISQ-12) of nulliparae during pregnancy.

**Methods:**

This is the second analysis of the baseline data from a randomized controlled trial (RCT), which aimed to study the protective efficacy of pelvic floor muscle training on stress urinary incontinence at 6th week postpartum (registration number: ChiCTR2000029618). Nulliparae aged 20–40 years with singleton pregnancy before 16 weeks of gestation were enrolled in this study, and data, including participants' demographic information, the Modified Oxford Scale (MOS), and PISQ-12, were collected. Eligible nulliparae were divided into two groups: Group MOS > 3 and Group MOS ≤ 3. Demographic information of the two groups was compared. Sexual function based on the PISQ-12 scores of the two groups was compared. A comparison of the PISQ-12 scores between the two groups was calculated by the Mann–Whitney *U*-test using SPSS version 23.0.

**Results:**

A total of 735 eligible nulliparae were enrolled in this study. Along with MOS grading up, PISQ-12 scores tended to get lower. Of the 735 nulliparae, there were 378 and 357 participants included in Group MOS > 3 and Group MOS ≤ 3, respectively. The PISQ-12 scores of Group MOS > 3 were significantly lower than those of Group MOS ≤ 3 (11 vs. 12, *p* < 0.001). The scores of the frequency of feeling sexual desire, orgasm achievement, sexual excitement, sexual activity satisfaction, sexual intercourse pain, fear of urinary incontinence, and negative emotion reactions with the sexual intercourse of Group MOS > 3 were lower than those of Group MOS ≤ 3 (*p* < 0.05).

**Conclusion:**

Pelvic floor muscle strength was positively associated with sexual function based on the questionnaire of young nulliparae during their first trimester. Up to half of the nulliparae during the first trimester were suffering from weak pelvic floor muscle strength and nearly a quarter of the nulliparae were facing this weakness combined with sexual dysfunction.

**Trial registration:**

This study has been registered at http://www.chictr.org.cn (registration number: ChiCTR2000029618).

## 1. Introduction

Female sexual dysfunction (FSD) encompasses various conditions that are reported as personal distress in one or more of the following areas: desire, arousal, orgasm, or pain (1). The prevalence of FSD ranges from 38 to 85.2%, which might be underestimated due to the low rate of complaint expressed by women regarding sexual experience and the help-seeking behavior displayed by them (2). The most common female sexual problem is the lack of interest in sex (34%) (3), while other commonly reported problems were the lack of orgasm achievement (16%) and the presence of vaginal dryness (13%) (3).

Female sexual experience is a vital part of life at any age and may be influenced by many factors. Some studies have demonstrated a strong positive correlation between sexual function and pelvic floor muscle strength (PFMS) (4–7). Consequently, investigators made various efforts to search for methods to improve the PFMS. Carcelén-Fraile proposed that PFM training (PFMT) can improve women's sexual function (8, 9). However, Baytur indicated that there was no confirmative association between sexual function and PFMS (10).

It is well-known that pregnancy and delivery are particularly recognized as important factors that have a negative influence on a woman's sexual life (11, 12). Controversial opinions regarding the correlation of sexual function with PFMS may be related to aging, parity, and gestation stages. Nulliparae are a special population who are more likely to not have confounding factors caused by delivery. Therefore, we aimed to explore the association of sexual function based on the Pelvic Organ Prolapse/Urinary Incontinence Sexual Questionnaire (PISQ-12) and the PFMS of nulliparae in the first trimester.

There was a multicenter randomized controlled trial, which was recently conducted by the Peking University People's Hospital (PKU-PH), that explored the protective efficacy of pelvic floor muscle training on stress urinary incontinence at the 6th week postpartum (registration number: ChiCTR2000029618) (13). We hypothesized that the pelvic floor muscle strength is irrelevant to sexual function based on the PISQ-12 scores during pregnancy. We tested this hypothesis by comparing participants' demography, PFMS, and PISQ-12 scores collected from nulliparae aged 20–40 years within 16 gestation weeks.

## 2. Materials and methods

This is the second analysis of the data from the multicenter, randomized controlled trial entitled “Effect of the App-Based Video Guidance on Prenatal Pelvic Floor Muscle Training Combined with Global Postural Re-Education for Stress Urinary Incontinence Prevention” (The Trial). The Trial design and characteristics of the patients have been published previously (13). The Trial was conducted by the PKU-PH and enrolled eight collaborative hospitals in China as the study sites from August 2020 to November 2022. The study was reviewed and approved by the Ethics Committee of the leading hospital—PKU-PH (IRB number: 2019PHD107-02) and was also approved by the ethics committees of the other eight participating study sites. Informed consent was obtained from each participant at each center in the study.

### 2.1. Participants

Participants were all recruited from the departments of obstetrics and gynecology of the nine hospitals. Nulliparae who were 20–40 years old with their first singleton pregnancy before 16 gestation weeks were eligible for enrollment in the trial. Participants were excluded from the trial, if they were (1) having serious adverse maternal or fetal complications (including brain, lung, heart, liver, kidney, and mental illness; coagulation dysfunction; or obvious cognitive dysfunction), (2) suffering from urinary incontinence, pelvic floor organ prolapse, or other pelvic floor dysfunction, (3) having a history of cervical insufficiency or recurrent miscarriage, (4) unable to complete all follow-up visits, (5) infected by coronavirus disease 2019 (COVID-19), and/or (6) unable to provide informed consent.

### 2.2. Working flow of this analysis

After thorough counseling and signing the informed consent forms, all participants took part in a series of self-administered questionnaires, including the Pelvic Organ Prolapse/Urinary Incontinence Sexual Questionnaire (PISQ-12) (14), and filled out the demographic form during the enrollment visit under the instructions of the site research assistants who were present in every study center. To ensure quality control, these assistants were blinded and allowed to assist the participants to understand the meaning of each and every question by interpretation but were not allowed to provide any decisions or inducing words to the participants so as to avoid tendency bias. After completing the questionnaires, the participants were led to an examination room for the assessment of the Modified Oxford Scale (MOS) and for undergoing other physical examinations.

### 2.3. Measurement

The demographic form, including the participant's age, pregestational body mass index (BMI; within 1 month before pregnancy), gestation weeks, educational status, occupational category, working posture, defecation posture, constipation history, smoking history, and regular PFM training (PFMT), was collected at the enrollment of the participants. To distinguish different age stages, we divided every 5 years from 20 to 40 years old into one category. According to the Chinese classification, BMI values of <18.5 kg/m^2^, 18.5 ≤ BMI <24 kg/m^2^, 24 ≤ BMI <28 kg/m^2^, and BMI≥28 kg/m^2^ represent underweight, normal weight, overweight, and obesity, respectively (15). Defecation posture refers to the posture that a woman adopted for most of her defecations and is categorized according to the potential impact on the PFM as squatting posture, sitting posture, or not specified (16). Regular PFMT here refers to performing PFMT at least one time a week for 20 min in total in the past 3 months. Occupational categories include low occupational activity (e.g., technicians and related support occupations, writers, artists, and management-related occupations), intermediate occupational activity (e.g., supervisors and proprietors, sales occupations, private household occupations, and health service occupations), and high occupational activity (e.g., farm and nursery workers, cleaning and building service occupations, and waitresses) (17).

The Pelvic Organ Prolapse/Urinary Incontinence Sexual Questionnaire was used to evaluate the sexual function in The Trial. PISQ-12 is a validated, objective, and self-administered questionnaire used for evaluating sexual function based on 12 questions, which identifies three distinct and separate domains of sexual function: behavioral emotive domain (Questions 1–4), physical domain (Questions 5–9), and partner-related domain (Questions 10–12). The scores were calculated by totaling the scores for each question with 0 = never and 4 = always. Reverse scoring was used for Questions 1–4. The total score (0–48) for a specific participant was used to evaluate her sexual function, with a lower score indicating better sexual function.

Data regarding the MOS were collected by the urogynecologists who were trained by the primary investigator (PI) from the PKU-PH. Each participant was examined by two urogynecologists and their scales were unified as one entry in the medical record. MOS is a widely accepted indicator in the evaluation of PFMS. It has been acknowledged that pregnant women in their first trimester have a higher risk of miscarriage than in the second and third trimesters. We did not apply the perineometer to evaluate the PFM function in pregnant women so as to avoid bringing them for examination as they were additionally anxious about the potential harm this procedure may cause to the fetus while inserting the perineometer into the vagina.

**Figure 1:**
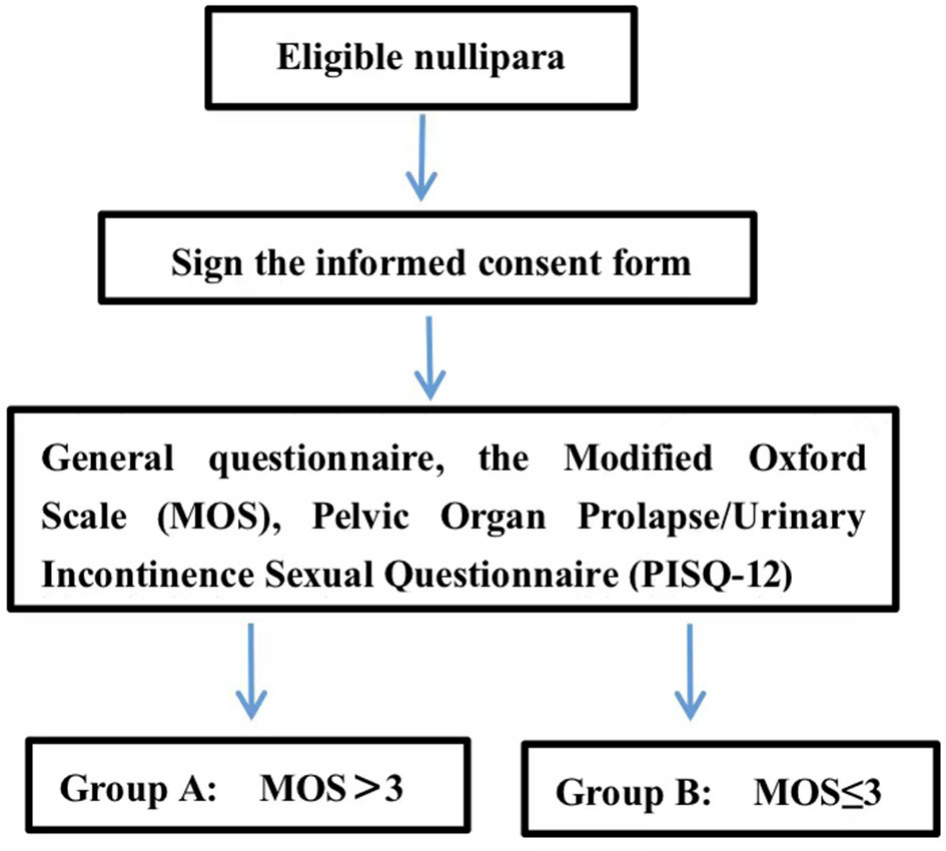
Study flow.

The Modified Oxford Scale was measured by a urogynecologist via vaginal palpation. The urogynecologist inserted their middle and index fingers into the participant's vagina and instructed her to squeeze and lift the fingers up. To avoid the involvement of the muscles of the hips, thighs, and abdomen during the transvaginal examination, the urogynecologist instructed the participant to contract the PFM subjectively without simultaneous co-contraction on other body parts. When the examination began, an investigator would put their hands on the participants' hips, thighs, and abdomen to confirm no sensible co-contraction from those parts. If the participant could not contract the PFM involuntarily, she would be guided to perform the motion of interrupting urination to help her feel PFM contraction. With verbal encouragement, the urogynecologist would request the participant to contract the PFM maximally and, at the same time, feel the pressure from the PFM against the fingers to grade the MOS. MOS would be graded as Grade 0 if no muscle contraction was felt, and Grade 1, 2, 3, 4, or 5 if muscle flicker or pulsation, weak muscle contraction, moderate contraction, good muscle contraction, or strong muscle contraction, respectively, was felt (18). In referring to the previous studies (19, 20), participants with MOS > 3 were included in Group MOS > 3, and others with MOS = 0, 1, 2, or 3 were included in Group MOS ≤ 3 (Figure 1). To ensure consistency among centers, all the urogynecologists who were assigned to conduct MOS grading were trained by the primary investigator (PI) in the PKU-PH.

### 2.4. Sample size

This analysis is based on the data from the previously described “The Trial” that had been introduced in our previous publication (13), in which the sample size was calculated using PASS 2019 software by a professional statistician according to the sample number that is needed to obtain enough statistical power for obtaining an answer to the study hypothesis. The sample size was determined to be at least 734 participants with complete data.

### 2.5. Statistical analysis

Statistical analysis was calculated by SPSS version 23.0 (SPSS, Inc., Chicago, IL, USA), and the assumed two-sided *p*-value was 5%. Categorical variables were described as numbers and percentages. When continuous variables followed a normal distribution, data were described as the means ± standard deviations (*SDs*). Otherwise, the medians (*P25, P75*) were calculated if continuous variables did not follow a normal distribution.

Multivariate regression analyses were performed to determine the related factor of sexual function by ordinal logistic regression analysis. The results were presented as odds ratio (OR), adjusted OR, and 95% confidence intervals (CIs). The score of each question in the PISQ-12 was compared by the Mann–Whitney *U*-test between the two groups.

## 3. Results

In total, 735 nulliparae (the participants) were included in this analysis. The participants' mean age was 30.18 years and their median *(P25, P75)* gestational weeks were 12 (11–13). The median *(P25, P75)* PISQ-12 score was 11 (9–14), and the median MOS grade was 4 (2–4). Table 1 shows the demographic data and the MOS distribution for all participants.

**Table 1:** The demographic data and the MOS distribution for all participants.

	**All participants**
**Age (years)**	
20–24	32 (4.4%)
25–29	243 (33.1%)
30–34	373 (50.7%)
35–40	87 (11.8%)
**BMI (kg/m** ^2^ **)**	
<18.5	91 (12.4%)
18.5–23.9	526 (71.6%)
24–27.9	89 (12.1%)
≥28	29 (3.9%)
**Educational status**	
Graduate degree	160 (21.8%)
Undergraduate	525 (71.4%)
High school	36 (4.9%)
Secondary education	14 (1.9%)
**Occupational category**	
Low occupational activity	465 (63.3%)
Intermediate occupational activity	250 (34.0%)
High occupational activity	20 (2.7%)
**Working posture**	
Sitting-posture	615 (83.7%)
Standing-posture	71 (9.7%)
Not specified	49 (6.7%)
**Defecation posture**	
Sitting-posture	588 (80%)
Squatting-posture	134 (18.2%)
Not specified	13 (1.8%)
**Constipation history**	
Yes	67 (9.1%)
No	668 (90.9%)
**Smoking history**	
Yes	11 (1.5%)
No	724 (98.5%)
**Regular PFMT**	
Yes	32 (4.4%)
No	703 (95.6%)
**MOS**	
Grade 0	6 (0.8%)
Grade 1	31 (4.2%)
Grade 2	149 (20.3%)
Grade 3	171 (23.3%)
Grade 4	225 (30.6%)
Grade 5	153 (20.8%)

Data are n (%), unless otherwise specified.

BMI, body mass index; PFMT, pelvic floor muscle training; MOS, the Modified Oxford Scale.

**Figure 2:**
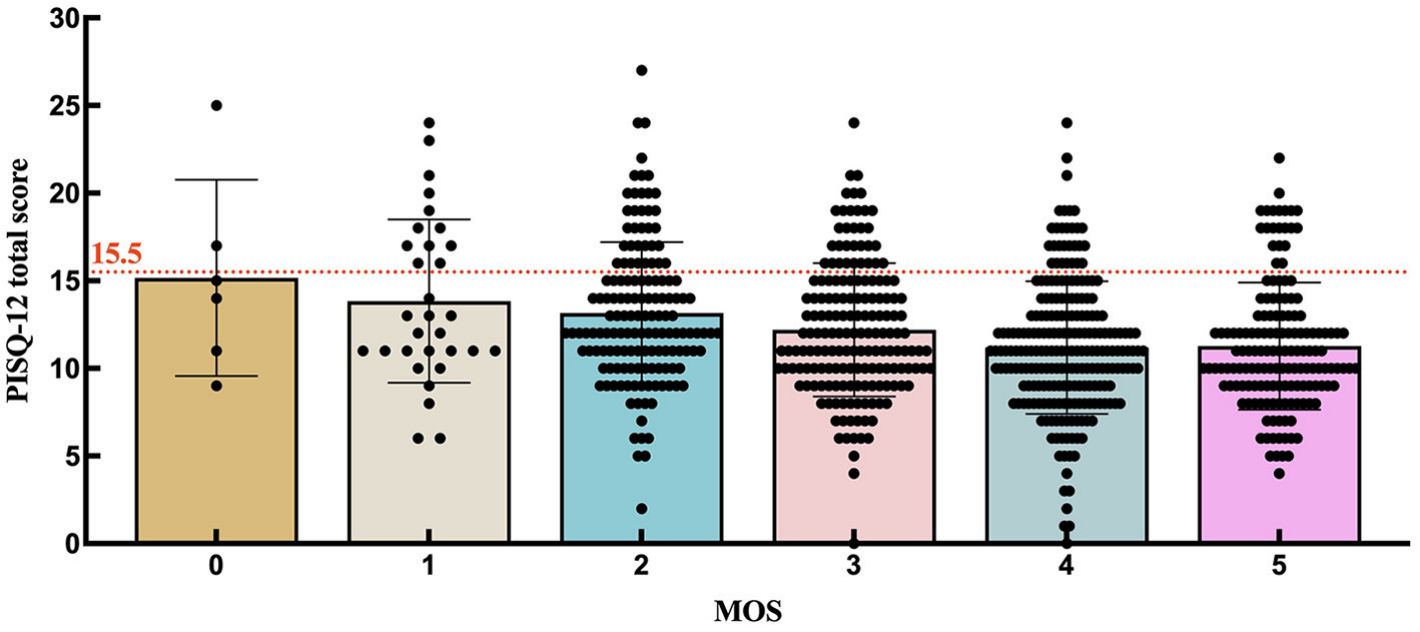
Distribution of PISQ-12 scores among participants with each MOS grade.

To further investigate the distribution of PISQ-12 scores among participants with each MOS grade, we prepared Table 2 and Figure 2. When setting the PISQ-12 score to >15.5 as the cutoff for FSD, as reported by Lau et al. (21), the percentage and number of participants with FSD among all with MOS = 0, =1, =2, =3, =4, and =5 were 33.3% (2/6), 38.7% (12/31), 24.2% (36/149), 19.3% (33/171), 12.9% (29/225), and 13.7% (21/153), respectively. Since there were only six women who were graded 0 for MOS, this result could not reflect the truth of women with MOS = 0. The proportion of FSD decreases from the participants with MOS = 1 to those with MOS = 4. As for the women with MOS = 5 who may make it complicated with a high PFM tone, the percentage of FSD is slightly higher than it is in women with MOS = 4. Figure 2 is supportive of Table 2 by showing the distribution of women below (normal sexual function) and above (FSD) the cutoff line with different MOS grades.

**Table 2:** The sexual function based on PISQ-12 scores for each MOS grade.

**MOS**	**PISQ-12 score**	**Normal sexual function**	**Sexual dysfunction**
Grade 0	15.17 ± 5.60^*^	4 (66.7%)	2 (33.3%)
Grade 1	13.84 ± 4.66^*^	19 (61.3%)	12 (38.7%)
Grade 2	12 (11–15)	113 (75.8%)	36 (24.2%)
Grade 3	12 (10–15)	138 (80.7%)	33 (19.3%)
Grade 4	11 (9–13)	196 (87.1%)	29 (12.9%)
Grade 5	11 (9–13)	132 (86.3%)	21 (13.7%)

Data are n (%), unless otherwise specified.

* Data are means ± standard deviations.

Age, BMI, constipation history, and regular PFMT are clinically recognized parameters that are associated with PISQ-12. Therefore, we performed an ordinal logistic regression analysis on the correlation of sexual function with MOS, age, BMI, constipation history, and regular PFMT (Table 3), which showed that none of these indicators were significantly altered when adding them to the logistic regression analysis one by one, with only an exception that adding MOS > 3 and MOS ≤ 3 created a significant difference in the results of the logistic regression analysis. Compared to MOS > 3, MOS ≤ 3 was significantly associated with lower sexual function (OR = 6.479; 95% CI: 3.387–12.393; *p* < 0.001).

**Table 3:** Ordinal logistic regression analysis of PISQ-12 score indicators.

	**OR**	**95% CI**	***P***
**Age (years)**			
20–24	1		
25–29	2.591	0.616–10.895	0.194
30–34	3.534	0.860–14.516	0.080
35–40	3.008	0.618–14.638	0.172
**BMI (kg/m** ^2^ **)**			
<18.5	1		
18.5–23.9	1.280	0.540–3.035	0.575
24–27.9	1.520	0.490–4.715	0.469
≥28	0.481	0.095–2.431	0.376
**Constipation history**			
No	1		
Yes	2.327	0.880–6.154	0.089
**Regular PFMT**			
Yes	1		
No	0.876	0.281–4.437-	1.116
**MOS**			
MOS > 3	1		
MOS ≤ 3	6.479	3.387–12.393	<0.001

PISQ-12, Pelvic Organ Prolapse/Urinary Incontinence Sexual Questionnaire.

Group MOS > 3 included 378 (51.43%, 378/735) participants and Group MOS ≤ 3 included 357 (48.57%, 357/735) participants. The PISQ-12 score of Group MOS > 3 was lower than that of Group MOS ≤ 3 (11 vs. 12, *p* < 0.001). The FSD percentage was significantly lower in Group MOS > 3 than in Group MOS ≤ 3 (13.2 vs. 23.2%, *p* < 0.001), indicating that the sexual function of participants with MOS > 3 was better than that of participants with MOS ≤ 3.

The behavioral emotive domain (Question 1–Question 4) of the PISQ-12 for all participants and univariate analysis results of the two groups are described in Table 4, which shows that the Group MOS > 3 was significantly lower than the Group MOS ≤ 3 in scores for each and the total of Questions 1–4 (*p* < 0.05), indicating that women with MOS > 3 were more frequent than those with MOS ≤ 3 in feeling sexual desires, orgasm achievement, sexual excitement, and sexual satisfaction, and those sex-related elements were significantly associated with PFMS.

**Table 4:** The behavioral emotive domain of PISQ-12 for all participants and univariate analysis results of the two groups.

	**All participants**	**Group MOS > 3 (*n* = 378, 51.43%)**	**Group MOS ≤ 3 (*n* = 357, 48.57%)**	***Z***	***P***
Q1. How frequently do you feel sexual desire? This feeling may include wanting to have sex, planning to have sex, feeling frustrated due to lack of sex, etc.	2 (2–2)^*^	2 (2–2)^*^	2 (2–3)^*^	−5.250	**<0.001**
0	12 (1.6%)	10 (2.6%)	2 (0.6%)		
1	93 (12.7%)	60 (15.9%)	33 (9.2%)		
2	458 (62.3%)	248 (65.6%)	210 (58.8%)		
3	131 (17.8%)	40 (10.6%)	91 (25.5%)		
4	41 (5.6%)	20 (5.3%)	21 (5.9%)		
Q2. Do you climax (have an orgasm) when having sexual intercourse with your partner?	2 (1–2)^*^	2 (1–2)^*^	2 (2–2)^*^	−3.427	**0.001**
0	36 (4.9%)	27 (7.1%)	9 (2.5%)		
1	176 (23.9%)	100 (26.5%)	76 (21.3%)		
2	398 (54.1%)	198 (52.4%)	200 (56.0%)		
3	94 (12.8%)	39 (10.3%)	55 (15.4%)		
4	31 (4.2%)	14 (3.7%)	17 (4.8%)		
Q3. Do you feel sexually excited (turned on) when having sexual activity with your partner?	2 (1–2)^*^	2 (1–2)^*^	2 (1–2)^*^	−3.847	**<0.001**
0	50 (6.8%)	28 (7.4%)	22 (6.2%)		
1	264 (35.9%)	158 (41.8%)	106 (29.7%)		
2	365 (49.7%)	173 (45.8%)	192 (53.8%)		
3	43 (5.9%)	13 (3.4%)	30 (8.4%)		
4	13 (1.8%)	6 (1.6%)	7 (2.0%)		
Q4. How satisfied are you with the variety of sexual activities in your current sex life?	1 (1–2)^*^	1 (1–2)^*^	2 (1–2)^*^	−3.077	**0.002**
0	95 (12.9%)	55 (14.6%)	40 (11.2%)		
1	276 (37.6%)	156 (41.3%)	120 (33.6%)		
2	290 (39.5%)	137 (36.2%)	153 (42.9%)		
3	52 (7.1%)	21 (5.6%)	31 (8.7%)		
4	22 (3.0%)	9 (2.4%)	13 (3.6%)		
Behavioral Emotive domain	7 (5–8)^*^	7 (5–8)^*^	8 (6–9)^*^	−5.132	**<0.001**

Data are n (%), unless otherwise specified.

* Data are median (P25, P75) of the question scores. The bold values indicate the value of *p* < 0.05.

Table 5 shows the correlation between PFMS and physical domain (Questions 5–9 of PISQ-12) for all participants and the results from univariate analysis of the two groups. Group MOS > 3 obtained a significantly lower score than Group MOS ≤ 3 for the total score for the physical domain and the score for the questions regarding sexual intercourse pains, fear of UI, and negative emotion reactions to sexual intercourse (*p* < 0.05). However, no significant difference was observed between the two groups in the scores for questions regarding the restriction of sexual activity due to urinary incontinence (UI) and pelvic organ prolapse (POP; *p* > 0.05).

**Table 5:** The correlation between PFMS and the physical domain of PISQ-12 for all participants and the results from univariate analysis of the two groups.

	**All participants**	**Group MOS > 3 (*n* = 378, 51.43%)**	**Group MOS ≤ 3 (*n*=357, 48.57%)**	***Z***	***P***
Q5. Do you feel pain during sexual intercourse?	1 (0–2)^*^	1 (0–1)^*^	1 (1–2)^*^	−2.452	**0.014**
0	197 (26.8%)	115 (30.4%)	82 (23.0%)		
1	351 (47.8%)	175 (46.3%)	176 (49.3%)		
2	154 (21.0%)	79 (20.9%)	75 (21.0%)		
3	27 (3.7%)	7 (1.9%)	20 (5.6%)		
4	6 (0.8%)	2 (0.5%)	4 (1.1%)		
Q6. Are you incontinent of urine (leak urine) with sexual activity?	0 (0–0)^*^	0 (0–0)^*^	0 (0–0)^*^	−0.136	0.891
0	662 (90.1%)	341 (90.2%)	321 (89.9%)		
1	65 (8.8%)	33 (8.7%)	22 (9.0%)		
2	7 (1.0%)	4 (1.1%)	3 (0.8%)		
3	1 (0.1%)	0	1 (0.3%)		
4	0	0	0		
Q7. Does fear of incontinence (either feces or urine) restrict your sexual activity?	0 (0–1)^*^	0 (0–1)^*^	0 (0–1)^*^	−2.890	**0.004**
0	506 (68.8%)	280 (74.1%)	226 (63.3%)		
1	101 (13.7%)	39 (10.3%)	62 (17.4%)		
2	71 (9.7%)	33 (8.7%)	38 (10.6%)		
3	28 (3.8%)	13 (3.4%)	15 (4.2%)		
4	29 (3.9%)	13 (3.4%)	16 (4.5%)		
Q8. Do you avoid sexual intercourse because of bulging in the vagina (either the bladder, rectum or vagina falling out)?	0 (0–0)^*^	0 (0–0)^*^	0 (0–0)^*^	−0.248	0.804
0	640 (87.1%)	328 (86.8%)	312 (87.4%)		
1	44 (6.0%)	22 (5.8%)	22 (6.2%)		
2	20 (2.7%)	14 (3.7%)	6 (1.7%)		
3	18 (2.4%)	7 (1.9%)	11 (3.1%)		
4	13 (1.8%)	7 (1.9%)	6 (1.7%)		
Q9. When you have sex with your partner, do you have negative emotional reactions such as fear, disgust, shame, or guilt?	0 (0–1)^*^	0 (0–0)^*^	0 (0–1)^*^	−2.326	**0.020**
0	528 (71.8%)	285 (75.4%)	243 (68.1%)		
1	145 (19.7%)	68 (18.0%)	77 (21.6%)		
2	57 (7.8%)	24 (6.3%)	33 (9.2%)		
3	3 (0.4%)	1 (0.3%)	2 (0.6%)		
4	2 (0.3%)	0	2 (0.6%)		
Physical domain	2 (1–3)^*^	2 (1–3)^*^	2 (1–4)^*^	−2.583	**0.010**

Data are n (%), unless otherwise specified.

* Data are median (P25, P75) of the question scores. The bold values indicate the value of *p* < 0.05.

Scores for the partner-related domain (Questions 10–12) of the PISQ-12 for all participants and the results from the univariate analysis of the two groups are shown in Table 6, which indicates that there were no significant differences between the two groups for the total and per-question scores for all those questions, except Question 12.

**Table 6:** The scores for the partner-related domain of PISQ-12 for all participants and the results from univariate analysis of the partner-related domain of PISQ-12 between the two groups.

	**All participants**	**Group MOS > 3 (*n* = 378, 51.43%)**	**Group MOS ≤ 3 (*n* = 357, 48.57%)**	***Z***	***P***
Q10. Does your partner have a problem with erections that affects your sexual activity?	0 (0–0)^*^	0 (0–0)^*^	0 (0–0)^*^	−0.811	0.417
0	602 (81.9%)	313 (82.8%)	289 (81.0%)		
1	111 (15.1%)	59 (15.6%)	52 (14.6%)		
2	20 (2.7%)	5 (1.3%)	15 (4.2%)		
3	2 (0.3%)	1 (0.3%)	1 (0.3%)		
4	0	0	0		
Q11. Does your partner have a problem with premature ejaculations that affects your sexual activity?	0 (0–0)^*^	0 (0–0)^*^	0 (0–0)^*^	−0.956	0.339
0	587 (79.9%)	307 (81.2%)	280 (78.4%)		
1	120 (16.3%)	58 (15.3%)	62 (17.4%)		
2	23 (3.1%)	11 (2.9%)	12 (3.4%)		
3	4 (0.5%)	2 (0.5%)	2 (0.6%)		
4	1 (0.1%)	0	1 (0.3%)		
Q12. Compared to orgasms you have had in the past, how intense are the orgasms you have had in the past 6 months?	2 (2–2)^*^	2 (2–2)^*^	2 (2–2)^*^	−2.462	0.014
0	22 (3.0%)	15 (4.0%)	7 (2.0%)		
1	20 (2.7%)	12 (3.2%)	8 (2.2%)		
2	605 (82.3%)	314 (83.1%)	291 (81.5%)		
3	79 (10.7%)	35 (9.3%)	44 (12.3%)		
4	9 (1.2%)	2 (0.5%)	7 (2.0%)		
Partner related domain	2 (2–3)^*^	2 (2–3)^*^	2 (2–3)^*^	−1.817	0.069

Data are n (%), unless otherwise specified.

* Data are median (P25, P75) of the question scores.

## 4. Discussion

The vast majority of the nulliparae in our study were in their first trimester at the age of 30.18 years with a median MOS of 4 (2–4). It is generally anticipated that young women in an early stage of pregnancy have not experienced delivery and should have normal PFMS. However, to our surprise, our result showed that 48.57% of participants with MOS ≤ 3 were those in the single-pregnant nulliparae within 16 gestation weeks. Although it has been proven that PFM could be weakened during pregnancy (22), the uterus produces a smaller force on PFM in the first trimester than in the second and third trimesters. According to relevant literature, the high ratio of weakened PFMS in this study may be associated with the alteration of hormones, such as relaxin that could potentially weaken PFM strength (23), and lack of physical exercise, core muscle training, and supervised PFM training (PFMT) before pregnancy (24, 25). A study on non-pregnant women aged 28.5 ± 3.6 years reported a mean MOS of 3.2 ± 0.9 (22), indicating that PFM weakness likely appears before pregnancy. Palmezoni also reported that the MOS of nulliparae in the first trimester aged 22.3 ± 5.5 years was 2.5, slightly lower than the PFM strength of non-pregnant women (22). Similarly, our study showed that a large number of young nulliparae experienced a weakening of PFMS in the first trimester. PFMT has been proven to be an effective method to improve pelvic floor function during pregnancy (26, 27). Therefore, we suggest that women perform PFMT under high-intensive supervision, including but not limited to the period of pregnancy.

Female sexual dysfunction (FSD) has a negative impact on women's emotional health and quality of life. However, many women tend to ignore their bad sense of sexuality, and this is especially obvious among Chinese women due to the influence of Chinese traditional culture. In fact, FSD is quite an important problem that is closely related to increases in divorce rates and domestic violence, which seriously damages family stability and even social stability (28). Thus, it was the duty of medical investigators to pay attention to the FSD.

Santos et al. (29) conducted a study to evaluate sexual function by the Female Sexual Function Index (FSFI) on 78 non-pregnant women aged 23 (15–38) years and 76 nulliparae aged 23 (20–30) years in the second trimester or more and obtained FSFI scores of 30.6 and 25.7, respectively. Their results demonstrated that sexual function in most of the non-pregnant women was normal, while it was slightly dysfunctional in most of the nulliparae in the second trimester or more. However, their study did not provide data regarding the sexual function of nulliparae in the first trimester. In our study, the median *(P25, P75)* score of the PISQ-12 in our participants was 11 (9–14), which, as reported by Lau et al. (21), represents a normal sexual function according to the cutoff of ≤ 15.5 for normal sexual function.

Many factors were proven to be associated with sexual function dysfunction (30), and PFM function was one of those factors. Zhuo et al. conducted a study involving 252 women aged 40–55 years and concluded that perimenopausal women with dysfunctional PFM had worse sexual function than those with functional PFM (28). Omodei analyzed the data of 226 sexually active women who had amenorrhea for >1 year and proposed that the PFMS in the perimenopause period was a significant factor in postmenopausal women's sexual function (6). In addition, other pieces of literature also found a similar conclusion in different age ranges (2, 4). However, Baytur et al., who recruited 68 participants having experienced vaginal delivery, cesarean delivery, and nulliparae for a study, proposed that there was no association between PFMS and the sexual function of all participants. Contradictory opinions among investigators may be related to the differences in their studies in the features of the participants and the sample sizes. Considering that aging and delivery are the known factors affecting the PFMS and sexual function (6), we recruited nulliparae aged 20–40 years to be the subjects of our analysis, to avoid the confounding bias related to aging and delivery. In the evaluation of the participants for sexual function via PISQ-12, we found that a better PFM strength is closely related to a better sexual function, especially in the behavioral emotive and physical domains. Strengthening pelvic floor muscles during pregnancy may be a suitable method to help women acquire better sexual satisfaction in the future.

Both the PISQ-12 and FSFI are questionnaires that were used for the evaluation of female sexual function and were validated for good concordance with each other for differentiating women with FSD from those without FSD (21). The Trial that our analysis is based on was designed to also evaluate the sexual function before and after delivery when women may have pelvic floor disorder (PFD) diseases, for which PISQ-12 is commonly known to be more suitable than FSFI. Higher PISQ-12 scores represent worse sexual function. Our analysis revealed that PFMS was inversely associated with the scores of behavioral emotive and physical domains and the total score of PISQ-12 (*p* < 0.05) was, therefore, positively related to sexual function. The strong correlation between PFMS and sexual function was especially observed in terms of the frequency of feeling sexual desire, orgasm achievement, sexual excitement, sexual activity satisfaction, sexual intercourse pain, fear of incontinence, and negative emotion during sexual intercourse. Those results are concordant with those of previous studies on non-pregnant women (4, 29).

The strengths of our analysis are its large sample size and multicenter implementation, which decrease the selection bias. In addition, the Trial could add new data on nulliparae in the first trimester for the association of pelvic floor muscle strength and sexual function. We hope that it can contribute to more specific clinical practice for young women.

However, our study has two limitations: (1) prepregnancy data from nulliparae were not collected as the comparable parameter of the evaluation, and (2) the electromyography examination of PFM was not adopted in getting objective data for the evaluation of PFM tone and strength. These limitations caused our analysis to only be able to reveal the correlation between PFM and female sexual function and unable to interpret the causality and further demonstrate if better PFM strength promotes better sexual function or the reverse. However, our analysis provides evidence for the necessity of high-quality RCTs for strengthening PFMS and improving the sexual function of young nulliparae.

## 5. Conclusion

Pelvic floor muscle strength was positively associated with sexual function based on the questionnaire of young nulliparae during their first trimester. Up to half of the nulliparae during the first trimester suffered from weak pelvic floor muscle strength and nearly a quarter of the nulliparae experienced this weakness combined with sexual dysfunction.

## Data availability statement

The original contributions presented in the study are included in the article/Supplementary material, further inquiries can be directed to the corresponding authors.

## Ethics statement

The studies involving human participants were reviewed and approved by the Ethics Committee of Peking University People's Hospital (IRB number: 2019PHD107-02). The patients/participants provided their written informed consent to participate in this study. Written informed consent was obtained from the individual(s) for the publication of any potentially identifiable images or data included in this article.

## Author contributions

LG and BX implemented this study and were responsible for data collection, analysis, and writing. LG supported the investigation and data analysis. HZ, DZ, BX, XF, HL, MZ, BQ, WC, XM, and LK were responsible for data collection. JW provided assistance in reviewing the manuscript. GW and XS guided the study design. All authors approved the final manuscript.
