# Supplementary material for: Association between pelvic floor muscle strength and sexual function based on PISQ-12—an analysis of data from a multicenter cross-sectional study on 735 nulliparae during pregnancy
Source: Front Med (Lausanne). 2023 Apr 25;10:1093830. doi: 10.3389/fmed.2023.1093830 (PMC10168537; doi:10.3389/fmed.2023.1093830)
Supplement: Supplementary file 1 [file Presentation_1.pdf]

Pelvic Organ Prolapse/Urinary Incontinence Sexual Function Questionnaire (PISQ-12)

Instructions: Following are a list of questions about you and your partner's sex life. All information is strictly confidential. Your confidential answers will be used only to help doctors understand what is important to patients about their sex lives. Please check the box that best answers the question for you. While answering the questions, consider your sexuality over the past six months. Thank you for your help.

1. How frequently do you feel sexual desire? This feeling may include wanting to have sex, planning to have sex, feeling frustrated due to lack of sex, etc.
   ☐ Always ☐ Usually ☐ Sometimes ☐ Seldom ☐ Never
2. Do you climax (have an orgasm) when having sexual intercourse with your partner?
   ☐ Always ☐ Usually ☐ Sometimes ☐ Seldom ☐ Never
3. Do you feel sexually excited (turned on) when having sexual activity with your partner?
   ☐ Always ☐ Usually ☐ Sometimes ☐ Seldom ☐ Never
4. How satisfied are you with the variety of sexual activities in you current sex life?
   ☐ Always ☐ Usually ☐ Sometimes ☐ Seldom ☐ Never
5. Do you feel pain during sexual intercourse?
   ☐ Always ☐ Usually ☐ Sometimes ☐ Seldom ☐ Never
6. Are you incontinent of urine (leak urine) with sexual activity?
   ☐ Always ☐ Usually ☐ Sometimes ☐ Seldom ☐ Never
7. Does fear of incontinence (either stool or urine) restrict your sexual activity?
   ☐ Always ☐ Usually ☐ Sometimes ☐ Seldom ☐ Never
8. Do you avoid sexual intercourse because of bulging in the vagina (either the bladder, rectum or vagina falling out?)?
   ☐ Always ☐ Usually ☐ Sometimes ☐ Seldom ☐ Never
9. When you have sex with your partner, do you have negative emotional reactions such as fear, disgust, shame or guilt?
   ☐ Always ☐ Usually ☐ Sometimes ☐ Seldom ☐ Never
10. Does your partner have a problem with erections that affects your sexual activity?
   ☐ Always ☐ Usually ☐ Sometimes ☐ Seldom ☐ Never
11. Does your partner have a problem with premature ejaculation that affects your sexual activity?
   ☐ Always ☐ Usually ☐ Sometimes ☐ Seldom ☐ Never

12. Compared to orgasms you have had in the past, how intense are the orgasms you have had in the past six months?
☐ Much less intense ☐ Less intense ☐ Same intensity ☐ More intense ☐ Much more intense

Scoring:

Scores are calculated by totaling the scores for each question with 0=never, 4=always. Reverse scoring is used for items 1,2,3 and 4. The short form questionnaire can be used with up to two missing responses. To handle missing values the sum is calculated by multiplying the number of items by the mean of the answered items. If there are more than two missing responses, the short form no longer accurately predicts long form scores. Short form scores can only be reported as total or on an item basis. Although the short form reflects the content of the three factors in the long form, it is not possible to analyze data at the factor level. To compare long and short form scores multiply the short form score by 2.58 (12/31).
